# Development and validation of a diagnostic model for late-onset neonatal sepsis using the haematological profile: a retrospective cohort study

**DOI:** 10.1007/s44253-026-00121-9

**Published:** 2026-05-06

**Authors:** Emily Hyde, Janno Schouten, Jarob Saker, H. R. Taal, H. Russcher, Eliane Wenstedt, Stephen Kennedy, Manu Vatish, Mark Anthony

**Affiliations:** 1 Nuffield Department of Women’s and Reproductive Health, University of Oxford, Oxford, OX3 9DU UK; 2 Division of Neonatology, Department of Neonatal and Paediatric Intensive Care, Erasmus University Medical Center, Rotterdam, The Netherlands; 3 Medical Scientific Department, Sysmex Europe, Norderstedt, Germany; 4 Department of Clinical Chemistry, Erasmus University Medical Center, Rotterdam, The Netherlands; 5 Neonatal Intensive Care Unit, Department of Paediatrics, Oxford University Hospitals NHS Trust, Oxford, UK

**Keywords:** Haematology, Neonatal sepsis, Blood cell count, Rapid diagnostic tests

## Abstract

**Purpose:**

Neonates admitted to intensive care units are at high risk of hospital-acquired infections. While blood cultures remain the gold standard for sepsis diagnosis, they do not detect infection in every case. Host immune response markers, such as C-reactive protein and procalcitonin, can assist in earlier detection but also have limitations and carry additional costs. We have developed a Neonatal Intensive Care Infection Score (NICIS), which leverages extended complete blood count (CBC+Diff + EIP) parameters, which may require specific analyser configuration or software licensing, to extract additional diagnostic value.

**Methods:**

We conducted a retrospective cohort study of neonates admitted to the Neonatal Intensive Care Unit at the John Radcliffe Hospital (Oxford, UK) over one year. Eighteen CBC+Diff + EIP parameters were initially considered based on their ability to differentiate culture-positive patients from those without clinical suspicion of infection. NICIS was developed as a weighted score and validated internally using a temporally distinct dataset and externally using a four-year retrospective cohort from Erasmus University Medical Center (Rotterdam, The Netherlands).

**Results:**

Incorporating eight CBC+Diff + EIP parameters, NICIS values ranged from 0 to 25, with higher scores indicating a greater likelihood of sepsis. NICIS achieved an area under the curve of 0.906 in training, 0.879 in internal validation, and 0.841 in external validation, outperforming C-reactive protein and total white blood cell count in all datasets.

**Conclusions:**

NICIS provides an interpretable tool for sepsis detection using routinely collected CBC+Diff + EIP data without additional sampling. Its performance across internal and external cohorts suggests sufficient diagnostic accuracy, although prospective studies are warranted to further assess generalisability and clinical utility.

**Supplementary Information:**

The online version contains supplementary material available at 10.1007/s44253-026-00121-9.

## Introduction

Neonatal sepsis remains a major contributor to infant mortality and long-term morbidity worldwide. In 2019, it was estimated to affect over 6.31 million babies and caused more than 200,000 deaths globally [1]. Preterm neonates, of whom approximately 15 million are born each year [2], are particularly vulnerable due to immature immune systems and frequent exposure to invasive procedures, such as central venous lines. In many high-income settings, approximately one in seven neonates is admitted to Neonatal Intensive Care Units (NICUs) annually.

Current international diagnostic guidelines, including those from the European Medicines Agency and World Health Organisation, recommend blood cultures as the diagnostic gold standard [3–5]. However, this approach has recognised limitations, including turnaround times of 24–48 h and variable sensitivity, particularly in neonates where small sample volumes and low-level bacteraemia are common [6]. C-reactive protein (CRP) is often used to monitor suspected infection, but its characteristic delay in rising, typically 12–24 h after infection onset, limits its early diagnostic utility [7]. While other biomarkers, such as procalcitonin and interleukins (particularly IL-6), may allow early detection of neonatal sepsis [8], they are not universally measured in routine clinical practice and are not included in all diagnostic guidelines. These limitations highlight the need for a rapid, reliable, and minimally invasive screening tool that complements, rather than replaces, diagnostic cultures and can be applied with minimal patient burden.

Automated haematological analysers routinely generate a conventional complete blood count (CBC), including red cell indices, total white cell count, and platelet count. Most laboratories also report a white cell differential (Diff), providing the proportions of neutrophils, monocytes, lymphocytes, basophils, and eosinophils (CBC+Diff). Beyond these standard outputs, modern analysers can also generate extended inflammatory parameters (EIP), which may provide additional insight into early immune responses (CBC+Diff + EIP) [9]. However, the ability to report these additional parameters may depend on analyser configuration and the availability of specific software licenses.

In adult sepsis, diagnostic models leveraging haematological parameters have shown promise. For example, the Intensive Care Infection Score (ICIS) uses five CBC+Diff + EIP parameters to distinguish sepsis from systemic inflammation, with reported sensitivity of up to 82.9% and specificity of up to 75.1% [10–12]. While models specific to neonatal sepsis exist, they are often complex and less interpretable. For example, a random forest model using 60 haematological and nine clinical variables achieved 88.0% specificity but only 38.0% sensitivity, limiting its utility for early detection [13]. There remains a need for an interpretable, implementable haematological model that generalises across diverse neonatal populations.

To address these gaps, we aimed to develop and externally validate a diagnostic model for neonatal sepsis using only haematological parameters, including those derived from CBC+Diff + EIP analysis. By improving early recognition, such a model could significantly improve outcomes in this highly vulnerable population.

## Methods

### Patient recruitment

This retrospective cohort study included all neonates admitted to the NICU at the John Radcliffe Hospital (Oxford, UK) between January 2022 and January 2023 who had a CBC+Diff + EIP measured during admission. The study used routinely collected, anonymised clinical data accessed under an existing approval from the Oxford Research Ethics Committee (REC 08/H0606/139), which permits secondary analysis of neonatal clinical data. An opt-out consent process was used, whereby parents were informed through patient information leaflets and consent was presumed unless explicitly declined. Consent could be withdrawn at any point without affecting care. All data were anonymised before analysis.

For external validation, an independent dataset was obtained from Erasmus University Medical Center (Rotterdam, The Netherlands). This cohort included preterm neonates born before 32 weeks’ gestation and admitted for at least three days between January 2018 and October 2021. These data were accessed under an existing institutional ethical approval that permitted the secondary use of anonymised clinical data for research purposes (MEC-2020-0584). The dataset was not collected specifically for this study and was used to assess the generalisability of NICIS.

### Data collection

In the internal dataset, CBC+Diff + EIP results from routine clinical care were obtained from two Sysmex (Kobe, Japan) analysers: an XN-2000 in the haematology laboratory and the XN-450 in the NICU. Raw data files were extracted directly from the analysers to ensure completeness, as not all parameters were automatically exported to the electronic patient records. Both analysers were calibrated and quality controlled according to manufacturer protocols. The XN-450 had previously undergone internal validation against the laboratory XN-2000 as part of standard commissioning audits before entering clinical use. Nucleated red blood cell (NRBC) counts from the XN-450, which lack external validation, were internally validated. White blood cell (WBC) and lymphocyte counts were adjusted accordingly for the present study.

The extended CBC result (CBC+Diff + EIP) included white cell differential, reticulocyte characterisation, and advanced inflammation markers such as neutrophil reactivity index (NEUT-RI) and neutrophil distribution width (NEUT-TW), which quantify cellular activation and heterogeneity in response to infection [14]. These parameters are automatically generated and do not require additional blood sampling; however, access to specific parameters depends on analyser configuration and licensing.

Matched clinical data were extracted from both paper and electronic health records, with paper charts providing hourly clinical observations and electronic records providing longitudinal clinical and laboratory data. Clinical data, such as antibiotic treatments, were recorded per day of admission. Where multiple CBC+Diff + EIP or other laboratory results were available for the same day, values were summarised using the mean. This approach avoided over-weighting days with repeated sampling while retaining clinically meaningful within-day variation.

In the external validation dataset, CBC+Diff + EIP results were measured using an XN-2000 analyser, also calibrated and quality controlled per the manufacturer’s protocols. Matched clinical data were extracted from electronic health records. Continuously monitored physiological parameters (e.g. heart rate) used to assess clinical deterioration were averaged hourly.

### Data categorisation

For both cohorts, CBC+Diff + EIP results that met the following criteria were excluded: within the first three days of life, when haematological parameters are physiologically unstable; two days following surgery, as this can trigger non-infective inflammation; and two days following transfusion, as these may have a general impact on cell counts.

**Fig. 1:**
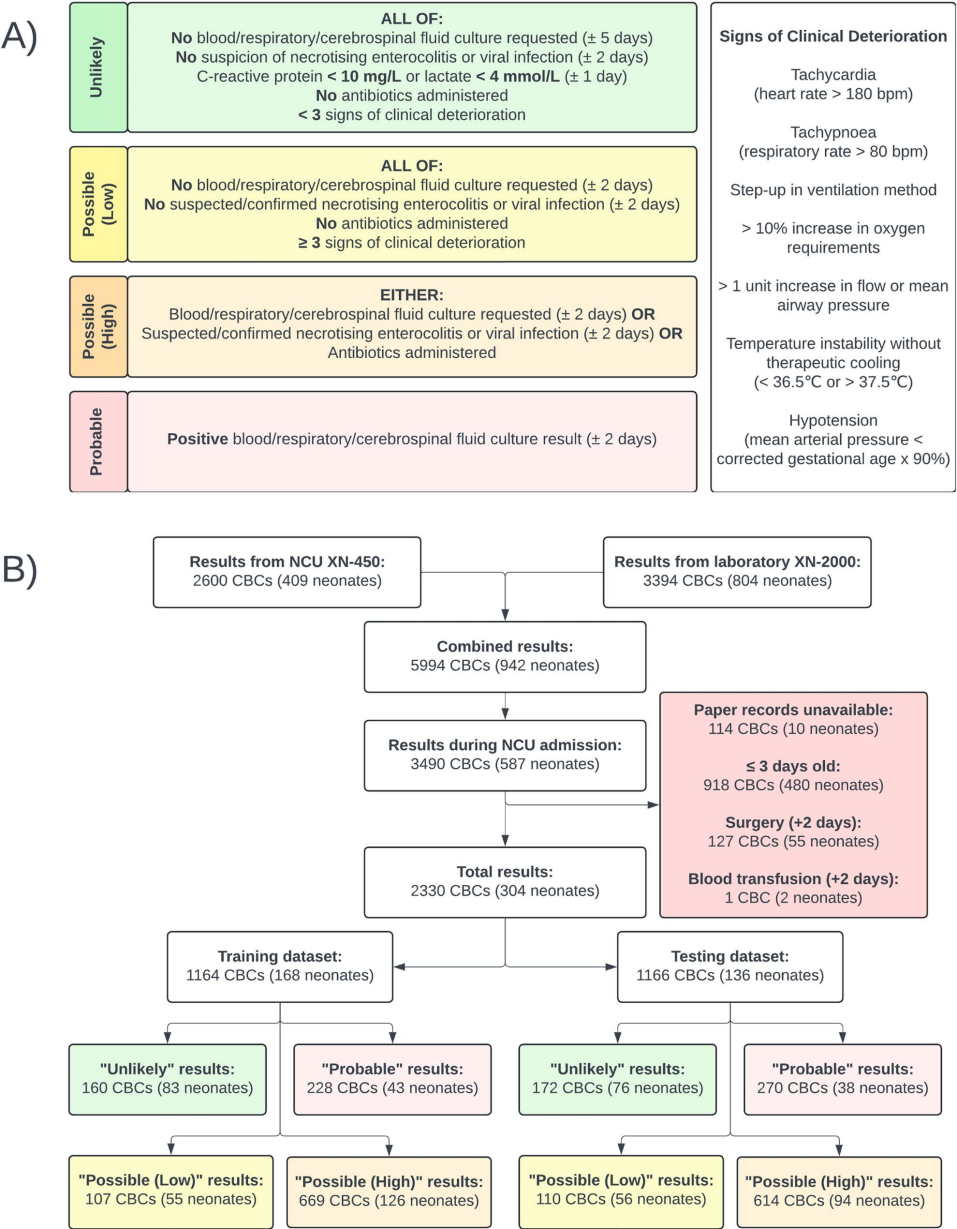
Categorisation and flowchart of inclusion of neonatal complete blood count (CBC) results for this study. (**A**) Categorisation of CBC+Diff + EIP results used in diagnosing neonatal sepsis. Signs of clinical deterioration were assessed daily using hourly physiological measurements from clinical observation charts. Each criterion was considered positive if at least one abnormal value occurred within the 24-hour period (e.g., heart rate > 180 bpm for tachycardia. (**B**) Flowchart for the inclusion and exclusion of CBC+Diff + EIP results used for developing a diagnostic model for neonatal sepsis

CBC+Diff + EIP results were categorised as defined in Fig. 1A. Cases (*probable sepsis*) were characterised by a positive blood, respiratory, or cerebrospinal fluid culture, consistent with NICE diagnostic standards for neonatal sepsis [3]. Per local guidelines, cultures were only requested when there was a clinical suspicion of sepsis and antibiotics were administered. Controls (*unlikely sepsis*) had no clinical suspicion of infection, no antibiotic treatment, normal CRP (less than 10 mg/L), and no culture requests, in line with local and published definitions [3].

CBC+Diff + EIP results not meeting either definition were classified as *possible (low or high) sepsis* and represented increasing levels of clinical suspicion, corresponding to physiological deterioration without intervention and escalation of care, respectively. These intermediate groups were excluded from model development but used to assess performance across a spectrum of sepsis suspicion.

The internal cohort was divided into training and testing datasets, stratified by sex, gestational age at birth, and total length of NICU stay.

### Parameter selection

Statistical analyses were conducted using R (v4.3.0). A complete list of all R packages used for data processing and analysis, including version numbers and references, is provided in Supplementary Table 1. Within the training dataset, individual CBC+Diff + EIP parameters were compared between *unlikely* and *probable sepsis* groups using either the Wilcoxon rank-sum test or Student’s t-test, based on underlying distributions (assessed using the Shapiro-Wilk test, *p* < 0.05).

ROC analysis was used to calculate the area under the curve (AUC), optimal thresholds based on Youden’s index, and other diagnostic performance metrics. Parameters were selected based on biological relevance and statistical significance, and to minimise redundancy. Candidate parameters with established or plausible roles in sepsis were prioritised [14], and those without significance in the training dataset (Student’s t-test or Wilcoxon rank-sum *p* > 0.05) were excluded. Where both absolute and relative counts were available, absolute counts were preferentially retained to reduce confounding. CBC+Diff + EIP results with missing values for any selected parameters were excluded from model development.

### Neonatal intensive care infection score development

A scoring system analogous to ICIS [10], referred to as the Neonatal Intensive Care Infection Score (NICIS), was developed using the training dataset. For each selected haematological parameter, three thresholds were derived from ROC analysis, and an AUC-weighted score was assigned as follows:

- Optimal threshold according to the Youden Index: 1 x AUC.
- Threshold achieving 85% specificity: 2 x AUC.
- Threshold achieving 95% specificity: 4 x AUC.

Thus, each parameter had three numeric cut-offs and three corresponding score levels. Parameters with greater individual diagnostic performance (i.e. larger AUCs) contributed proportionally higher scores. The final NICIS value for each CBC+Diff + EIP result was calculated as the sum of scores across all parameters, such that those exceeding higher thresholds contributed proportionally more to the overall score.

To refine the model, parameters were iteratively removed (from lowest to highest individual AUC), and the composite AUC recalculated to identify the optimal combination of parameters. Final performance metrics, including AUC, optimal threshold, sensitivity, and specificity, were derived using ROC analysis. An AUC greater than 0.8 and sensitivity, specificity, PPV, and NPV greater than 85% were considered indicative of strong diagnostic performance, chosen a priori based on consensus for clinically meaningful discrimination.

### Scoring system validation

NICIS was internally validated on the testing dataset and externally validated using the external validation dataset. Thresholds for sufficient diagnostic performance were predefined based on published benchmarks for neonatal sepsis biomarkers [7]. Specifically, an AUC greater than 0.8 and sensitivity, specificity, positive predictive value (PPV), and negative predictive value (NPV) greater than 85% were considered indicative of strong performance.

## Results

### Patient characteristics

During the study period, 3,490 CBC+Diff + EIP results were collected from 587 neonates admitted to the NICU at the John Radcliffe Hospital (Table 1; Fig. 1B). After excluding results within the first three days of life or two days following surgery or transfusion, 2,330 CBC+Diff + EIP results from 304 neonates met the inclusion criteria.

**Table 1:** Patient characteristics for the complete blood count (CBC) results included in this study. Median (IQR) values for gestational age at birth and sex were calculated from individual patient data, while postnatal age at sampling was calculated from all values. Values were compared using ^a^chi-square test, ^b^wilcoxon rank sum test, and ^c^wilcoxon signed rank test

	Training Dataset			Testing Dataset			Validation Dataset			Comparisons	
	All Values	Unlikely	Probable	All Values	Unlikely	Probable	All Values	Unlikely	Probable	Train vs. Test	Train vs. Validation
CBC+Diff + EIP Results	1164	160	228	1166	172	270	2638	1738	399	-	-
Neonates	168	83	43	136	76	38	474	412	142	-	-
CBC+Diff + EIP Results per Neonate	3 (1–8)	1 (1–2)	4 (3–6)	3 (1–10)	2 (1–3)	4 (2–11)	3 (1–7)	2 (1–5)	2 (1–3)	0.09^b^	0.83^b^
Gestational Age at Birth (Weeks)	32^+1^ (27^+3^–37^+4^)	29^+4^ (26^+2^–35^+1^)	26^+5^ (24^+4^–30^+3^)	31^+1^ (26^+6^–36^+2^)	29^+6^ (26^+5^–34^+4^)	26^+0^(24^+4^–28^+6^)	28^+0^(26^+2^–29^+5^)	28^+1^(26^+2^–29^+5^)	26^+4^ (25^+2^–28^+5^)	0.15^b^	< 0.01^c^
Sex (Males)	94 (56.0%)	46 (55.4%)	22 (51.2%)	80 (58.8%)	47 (61.8%)	25 (65.8%)	277 (58.4%)	243 (59.0%)	86 (60.6%)	0.70^a^	0.83^a^
Postnatal Age at Sampling (Days)	19 (9–37)	26 (11–48)	19 (10–33)	21 (10–40)	24 (10–52)	24 (14–43)	29 (12–68)	31 (13–71)	31 (13–65)	0.13^b^	< 0.01^c^

The cohort was divided into training and testing datasets using stratified sampling based on gestational age at birth, NICU stay duration, and sex. The training and testing datasets showed no statistically significant differences in gestational age at birth (*p* = 0.15), sex (*p* = 0.70), or postnatal age at sampling (*p* = 0.10), as shown in Table 1. Among CBC+Diff + EIP results classified as *probable sepsis* in the internal cohort, 45% (*n* = 222/498) were associated with a positive blood culture, while the remainder were defined based on positive respiratory cultures.

The external validation dataset comprised 2,638 CBC+Diff + EIP results from 182 neonates with significantly lower median gestational age (*p* < 0.01) and a higher postnatal age at sampling (*p* < 0.01). These differences reflect the inclusion criteria of the external cohort, which was limited to neonates born before 32 weeks’ gestation.

### Parameter selection

A total of 49 haematological parameters were evaluated in the training dataset. Of these, 43 showed statistically significant differences between the *unlikely* and *probable sepsis* groups (Supplementary Table 2). Thirteen parameters had an AUC greater than 0.700, indicating moderate to sufficient diagnostic accuracy. Additionally, 24 parameters had greater than 70.0% sensitivity, and 22 had greater than 70.0% specificity at their respective optimal thresholds.

The neutrophil-to-lymphocyte ratio (NLR) showed the highest performance, with 76.7% sensitivity, 76.3% specificity, and an AUC of 0.825, outperforming individual neutrophil and lymphocyte percentages. NEUT-RI also demonstrated strong diagnostic value (sensitivity: 75.0%; specificity: 74.4%; AUC: 0.812).

Based on these results, 18 parameters were selected for inclusion in the initial scoring system (Supplementary Table 2).

### Scoring system development and validation

After iteratively removing the parameters with the lowest individual AUCs, the final NICIS model incorporated eight CBC+Diff + EIP parameters (Table 2). For each parameter, the corresponding thresholds were determined from ROC analyses. Likelihood thresholds were derived from the training dataset corresponding to predefined sensitivity and specificity targets: a NICIS value of 2.255 corresponded to 95% sensitivity (*possible low*), 7.747 to 90% specificity (*possible high*), and 9.386 to 95% specificity (*probable*).

**Table 2:** The neonatal intensive care infection score (NICIS) for neonatal sepsis, based on complete blood count (CBC) parameters. Parameters are assigned scores equal to 1x, 2x, or 4x their area under the curve (AUC) based on three key thresholds. The final NICIS value is the sum of these scores. Abbreviations: ch – channel units from the scattergram; FI – fluorescence intensity; SI – scatter intensity

Parameter	Units	Direction	Optimal Threshold (1x AUC)	Threshold at 85% Specificity (2x AUC)	Threshold at 95% Specificity (4x AUC)	AUC
Reticulocyte haemoglobin equivalent (RET-HE)	pg	↓	29.6	26.0	23.1	0.703
Neutrophil count (NEUT#)	x10^9^/L	↑	5.46	6.39	8.68	0.796
Immature granulocyte count (IG#)	x10^9^/L	↑	0.19	0.30	0.70	0.745
Neutrophil lymphocyte ratio (NLR)	%	↑	1.46	1.80	2.52	0.825
Neutrophil reactive intensity (NEUT-RI)	FI	↑	46.1	48.0	51.2	0.812
Neutrophil true width (NEUT-TW)	ch	↑	37.3	40.0	55.1	0.811
Platelet count (PLT#)	x10^9^/L	↓	257	206	112	0.731
Immature platelet fraction (IPF)	%	↑	7.5	17.8	25.5	0.740

To calculate NICIS for a given CBC+Diff + EIP result, each parameter is assigned a score based on which threshold it crosses: 1 × AUC for the optimal threshold, 2 × AUC for the threshold achieving 85% specificity, and 4 × AUC for the threshold achieving 95% specificity. The individual parameter scores are then summed to calculate the total NICIS value. A worked example of this calculation is provided in Supplementary Table 3.

**Fig. 2:**
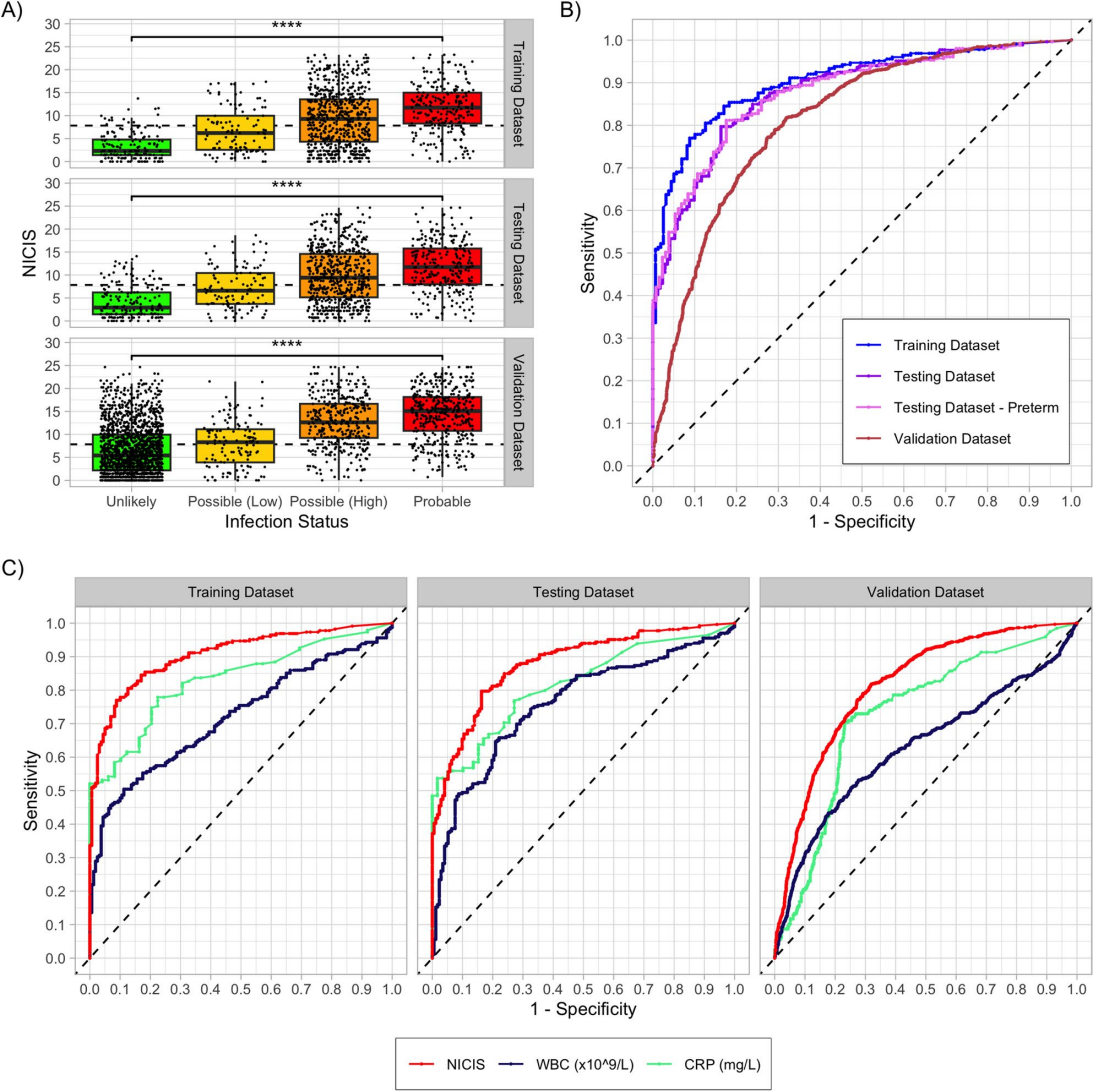
Diagnostic performance of the Neonatal Intensive Care Infection Score (NICIS) for neonatal sepsis. (**A**) Boxplots showing score distributions with clinical suspicion of sepsis across training, internal testing, and external validation datasets. (**B**) Receiver operating characteristic curves for each dataset. An additional ROC curve representing a subset of preterm neonates (< 32 weeks’ gestation) from the testing dataset is included as a visual comparison for the preterm-only external validation cohort. (**C**) Receiver operating characteristic curves for NICIS compared to C-reactive protein (CRP) and white blood cell (WBC) count. NB: CRP > 10 mg/L was an exclusion criterion for the unlikely sepsis (control) group

The model demonstrated consistently sufficient diagnostic accuracy across all three datasets, with AUCs of 0.906 (training), 0.879 (testing), and 0.841 (validation) (Fig. 2B). Comparison of AUCs using DeLong’s test showed no statistically significant difference between the training and testing datasets (DeLong’s *p* = 0.23). In contrast, performance was lower in the external validation dataset (DeLong’s *p* < 0.001 versus training). As shown in Fig. 2A, NICIS values increased proportionally with clinical suspicion. Performance metrics, including sensitivity and specificity, are summarised in Table 3.

**Table 3:** Diagnostic performance of the Neonatal Intensive Care Infection Score (NICIS) for late-onset neonatal sepsis. Key performance indicators include area under the curve (AUC), true positives (TP), true negatives (TN), false positives (FP), false negatives (FN), positive predictive value (PPV), negative predictive value (NPV), positive likelihood ratio (LR+), negative likelihood ratio (LR-), and diagnostic odds ratio (DOR), all presented with 95% confidence limits

	Training Dataset	Testing Dataset	Validation Dataset
AUC	0.906 (0.876–0.935)	0.879 (0.848–0.911)	0.841 (0.821–0.861)
Sensitivity	77.0% (70.9% – 82.3%)	77.8% (72.3% – 82.7%)	85.3% (81.4% – 88.7%)
Specificity	91.2% (85.7% – 95.1%)	83.7% (77.3% – 88.9%)	65.0% (62.7% – 67.2%)
TP	174	207	337
TN	145	144	1128
FP	14	28	608
FN	52	59	58
PPV	92.6% (87.8% – 95.9%)	88.1% (83.2% – 91.9%)	35.7% (32.6% – 38.8%)
NPV	73.6% (66.9% – 79.6%)	70.9% (64.2% – 77.1%)	95.1% (93.7% – 96.3%)
LR+	8.74 (5.28–14.49)	4.78 (3.39–6.75)	4.44 (2.26–2.63)
LR-	0.25 (0.20–0.32)	0.26 (0.21–0.33)	0.23 (0.18–0.29)
DOR	34.66 (18.46–65.07)	18.04 (10.97–29.68)	10.78 (8.02–14.49)
Youden	0.68 (0.57–0.77)	0.62 (0.50–0.72)	0.50 (0.44–0.56)
DeLong’s P-Value	-	0.23	< 0.001

An alternative model was developed that incorporated gestational age at birth and postnatal age at sampling. Although gestational age at birth was included in the final alternative model, no significant improvement in performance was observed compared to the CBC+Diff + EIP-only model (DeLong *p* = 0.64).

To facilitate comparison with the external validation cohort, which included only preterm neonates (less than 32 weeks’ gestation), an additional ROC curve was generated using a preterm subset of the testing dataset. This analysis was exploratory and intended to illustrate the impact of gestational age on NICIS performance rather than to provide a separate validation.

In a descriptive analysis of 126 paired NICIS-CRP measurements collected within ± 2 h on *probable sepsis* days, both NICIS and CRP exceeded their respective thresholds in 59 pairs (47%; Supplementary Table 4). NICIS exceeded its optimal threshold (7.84) while CRP remained below 10 mg/L in 46 pairs (37%), and CRP was abnormal while NICIS remained below threshold in 8 pairs (6%). These findings are descriptive and exploratory only. Illustrative case studies demonstrating NICIS trajectories during sepsis episodes are provided in Supplementary Fig. 1.

## Discussion

In this study, we developed and validated NICIS, a CBC-based tool for identifying neonatal sepsis. NICIS evaluates haematology parameters from routinely measured neonatal samples to detect host immune response patterns, without the need for additional sampling. Rather than replacing existing biomarkers, NICIS provides complementary information by capturing clinically relevant cellular changes associated with suspected infection.

Although some CBC+Diff + EIP parameters performed well individually (e.g. neutrophil count with an AUC of 0.796, 70.6% sensitivity, and 78.8% specificity), none were sufficient alone for early detection. This aligns with the NICE evidence review, which found limited support for the use of the WBC count, neutrophil count, and immature-to-total neutrophil ratio (ITR) in neonatal sepsis diagnostics [15]. These findings reinforce the value of integrating multiple CBC+Diff + EIP parameters.

The eight haematological parameters included in NICIS capture key aspects of the expected host response to infection. Neutrophil and immature granulocyte (IG) counts, NLR, NEUT-RI, and NEUT-TW reflect neutrophil activation and phenotypic heterogeneity, which are central to early sepsis responses [14]. NLR, in particular, is a well-supported marker of neonatal infection, reflecting shifts in neutrophil and lymphocyte populations during early immune activation [16, 17]. Platelet count and immature platelet fraction (IPF) reflect platelet activation and consumption, processes increasingly recognised in neonatal and adult sepsis [18]. Collectively, these neutrophil-platelet parameters align with the innate-dominant immune profile in neonates, where adaptive responses are slower to mobilise [19].

The ideal tool for neonatal sepsis diagnosis would achieve near-perfect sensitivity and PPV, and at least 85% specificity and NPV [7]. Recognising the limitations of real-world datasets, we considered an AUC greater than 0.8 and sensitivity, specificity, PPV, and NPV greater than 85% to represent strong diagnostic performance, consistent with predefined targets set a priori. NICIS met or exceeded these criteria in the training, testing, and external validation datasets, with AUCs of 0.906, 0.879, and 0.841, respectively. Specificity reached 91.2% in the training dataset while sensitivity was highest in the external validation dataset (85.3%), demonstrating the model’s generalisability.

The PPV in the external validation dataset was substantially lower (36%) than in the internal datasets (93% and 88%), reflecting the high proportion of *unlikely sepsis* cases and highlighting the importance of interpreting precision in the context of disease prevalence. Conversely, the higher sensitivity likely reflects the greater number of true positives in this cohort. The NPV in the external cohort was high (95%), suggesting potential utility for risk stratification. Differences observed in the preterm-only validation cohort may also reflect physiological differences in haematological profiles rather than model overfitting. These variations illustrate how class imbalances and prevalence influence practical performance metrics beyond AUC.

Alternative modelling approaches, including logistic regression and random forest, had similar performance but were less interpretable and transparent (data not shown). The random forest model, although most accurate, exhibited overfitting, required complex computational resources, and offered limited transparency. Similarly, the logistic regression model was interpretable but sensitive to outliers. In contrast, NICIS applies fixed, clinically meaningful thresholds across selected haematological parameters, improving interpretability and ease of implementation in settings where the required analyser configuration and licences are available.

NICIS was designed to be technically compatible with existing laboratory workflows, as it is derived from routinely generated CBC+Diff + EIP parameters. Laboratories with access to the required analyser outputs could calculate and report NICIS automatically with each CBC+Diff + EIP, enabling clinicians to use the score without additional sampling or procedural burden. A prototype online calculator (available at https://4fsqon-emily-hyde.shinyapps.io/NICISCalculator/) provides a practical interface for clinical or research use, facilitating external validation and real-time evaluation across diverse neonatal populations.

### Comparison with existing diagnostic systems

Haematological-based models have been previously explored for neonatal sepsis diagnosis. The Haematological Scoring System (HSS), developed by Rodwell et al. in 1988, used peripheral blood smears to assess abnormal counts and morphology [20]. More recently, the Sepscore attempted to modernise this approach but showed only modest diagnostic performance (68.0% sensitivity and 61.0% specificity) [21]. Similarly, machine learning models developed by Huang et al. demonstrate strong diagnostic accuracy in their respective cohorts but require complex inputs (60 CBC and nine clinical variables), limiting their clinical feasibility and implementation [13].

NICIS quantifies the host immune response to infection using a small number of haematological parameters obtained from routinely collected neonatal blood samples, without requiring additional blood sampling beyond the standard CBC. This is particularly relevant for preterm neonates, where blood volume is limited. Most parameters are automatically generated without requiring additional blood sampling beyond the standard CBC, although reticulocyte indices (RET-HE) and neutrophil activation markers (NEUT-RI, NEUT-TW) may require additional analyser licensing, depending on the region.

NICIS was conceptually informed by ICIS [10], which demonstrated that composite haematological scores could capture clinically meaningful host responses to infection. However, NICIS differs in design and application to reflect the neonatal intensive care setting, incorporating relevant haematological features and applying threshold-based weighting to prioritise interpretability and generalisability across heterogeneous neonatal populations. Unlike ICIS, which was developed in adult populations, NICIS was derived and validated exclusively in neonatal cohorts, including external validation across institutions.

### Strengths and limitations

A key strength of this study is the use of large, real-world datasets for both model development and validation. In addition to internal temporal validation, NICIS was evaluated on an external dataset with distinct demographic and clinical characteristics, supporting its generalisability. An alternative model that incorporated gestational age at birth did not outperform the CBC-only model, further confirming the robustness of our initial approach.

A post hoc evaluation of sample size adequacy was conducted per established guidelines for clinical prediction model development. Events per predictor (EPP) were calculated using the number of *probable sepsis* cases and the number of predictors retained in the final model. The final model incorporated eight predictors. In the training dataset, 228 CBC+Diff + EIP results were classified as *probable* sepsis, yielding an EPP of 29, while the testing and validation datasets achieved EPPs of approximately 34 and 50, respectively, exceeding the commonly recommended thresholds of 10–20 EPPs.

NICIS was designed for clinical applicability. Although more complex than single-parameter interpretation, each component has a defined biological rationale, and its contribution can be understood through individual AUC values, supporting transparency and interpretability. NICIS also demonstrated a graded increase in median score with increasing clinical suspicion (*unlikely*,* possible (low)*,* possible (high)*, and *probable sepsis*), despite only being trained on the extreme categories. This approach reduced noise from transient, non-sepsis-related fluctuations and ensured that controls reflected clinically stable neonates, while the observed gradient supports the model’s biological plausibility and generalisability.

To contextualise model performance, extreme NICIS values within the *unlikely* and *probable sepsis* groups were reviewed (data not shown). These exploratory reviews suggested that some discrepancies between NICIS values and clinical categories may reflect classification uncertainty, often due to contaminated cultures or incomplete clinical information. Because these observations were not quantified or included in the primary statistical analyses, they are reported only as potential contributors to variability rather than confirmed findings.

The main limitations of this study relate to its retrospective design and reliance on routinely collected data. Blood samples were obtained at the attending clinicians’ discretion, introducing potential selection bias and possibly underrepresenting stable neonates. To mitigate this, staff were encouraged to use the pre-dilution mode, enabling CBC sampling from 20 µL of capillary blood. Although results confounded by surgery or transfusions were excluded, unmeasured clinical factors may still have influenced the data, and missing or inconsistent values were likely present.

Where multiple CBC+Diff + EIP results were obtained from the same neonate on a single day, values were averaged to prevent repeated measurements from disproportionately influencing the model. This approach was applied only to the internal dataset, whereas the external dataset comprised single, timestamped CBC+Diff + EIP results. Nonetheless, daily averaging may have masked rapid, clinically relevant immune changes, underscoring a limitation inherent to retrospective datasets with irregular sampling intervals.

Another key limitation is the relatively low proportion of probable sepsis cases confirmed by positive blood cultures. In the internal cohort, many *probable sepsis* cases were defined by positive respiratory cultures. While this approach reflects clinical practice, it introduces uncertainty into case classification and may allow misclassification. Only the *unlikely* and *probable sepsis* groups were included in analyses, and neonates with confounding conditions such as NEC, viral infection, or surgical intervention were excluded.

A formal lagged analysis was not feasible because CBC+Diff + EIP and CRP results were measured at irregular intervals. Comparisons of paired NICIS-CRP results demonstrate that NICIS frequently exceeded its threshold even when CRP remained normal. However, given variable sampling intervals and limited information about the timing of infection onset, these observations are descriptive and should not be interpreted as indicating absolute lead time. Additionally, biomarkers such as procalcitonin and interleukins (particularly IL-6), which are used in some NICUs to enable earlier detection of sepsis, were not routinely measured in our training cohort and therefore could not be evaluated. If interpreted in isolation, elevated NICIS values could theoretically increase the risk of overdiagnosis or overtreatment, reinforcing the need to evaluate NICIS alongside clinical assessment and other diagnostic markers.

Finally, technical variability between haematology analysers represents a potential source of error. The XN-450 series reports NRBCs as lymphocytes, which may distort lymphocyte counts in neonatal samples. Paired neonatal samples from routine clinical care were previously used for internal verification between the XN-450 and the laboratory XN-2000. NRBC counts from the XN-450, which lack external validation, were internally verified, and WBC and lymphocyte counts were adjusted accordingly. These internal audits were conducted independently of this study, which remains fully retrospective.

These limitations highlight the need for prospective studies in well-characterised neonatal cohorts to further evaluate the performance, generalisability, and clinical impact of NICIS before routine implementation.

## Conclusion

We have developed and validated NICIS, a scoring system based on the extended neonatal CBC for the evaluation of neonatal sepsis, demonstrating sufficient diagnostic accuracy across both internal and external datasets. While NICIS is not a replacement for microbiological cultures, it provides an interpretable, automatable tool to quantify host immune activation.

### Take-home message

NICIS is a CBC-derived scoring system that quantifies neonatal immune activation and shows sufficient diagnostic accuracy across internal and external cohorts. When evaluated alongside current diagnostic tools, NICIS provides complementary information regarding immune activation without requiring additional blood sampling.

## Electronic supplementary material

Below is the link to the electronic supplementary material.

Supplementary Fig. 1: Case studies illustrating the longitudinal utility of the Neonatal Intensive Care Infection Score (NICIS) compared to C-reactive protein (CRP). (A) Case study of a neonate born at 30 weeks’ gestation and admitted to the John Radcliffe Neonatal Intensive Care Unit. On days 27, 28, and 29, their blood cultures tested positive for *Serratia marcescens*. (B) Case study of a neonate born at 26 weeks’ gestation and admitted to the Erasmus University Medical Center Neonatal Intensive Care Unit. On days 90 and 98, their blood cultures tested positive for *Klebsiella pneumoniae.* NICIS values (black) and CRP measurements (purple) with antibiotic administration (blue lines and dots), positive/negative blood cultures (red/black crosses), NICIS thresholds for 95% sensitivity and 95% specificity (dashed grey line), and the optimal NICIS threshold (solid grey line)

Supplementary Material 2

## Data Availability

The data underlying this study are not publicly available due to institutional and ethical restrictions related to patient confidentiality. De-identified datasets may be made available from the corresponding author upon reasonable request, subject to approval by the relevant institutional review boards and data sharing agreements. The analytical codes for this study are not publicly available but may be shared upon reasonable request. An interactive web-based version of the NICIS calculator has been produced using R Shiny and is available online at https://4fsqon-emily-hyde.shinyapps.io/NICISCalculator/ to facilitate clinical and research use. This tool is intended for research and educational purposes only, and the authors accept no responsibility for clinical use or outcomes.
