# Supplementary figures and images for: Development and validation of a diagnostic model for late-onset neonatal sepsis using the haematological profile: a retrospective cohort study
Source: Intensive Care Med Paediatr Neonatal. 2026 May 6;4(1):13. doi: 10.1007/s44253-026-00121-9 (PMC13314925; doi:10.1007/s44253-026-00121-9)

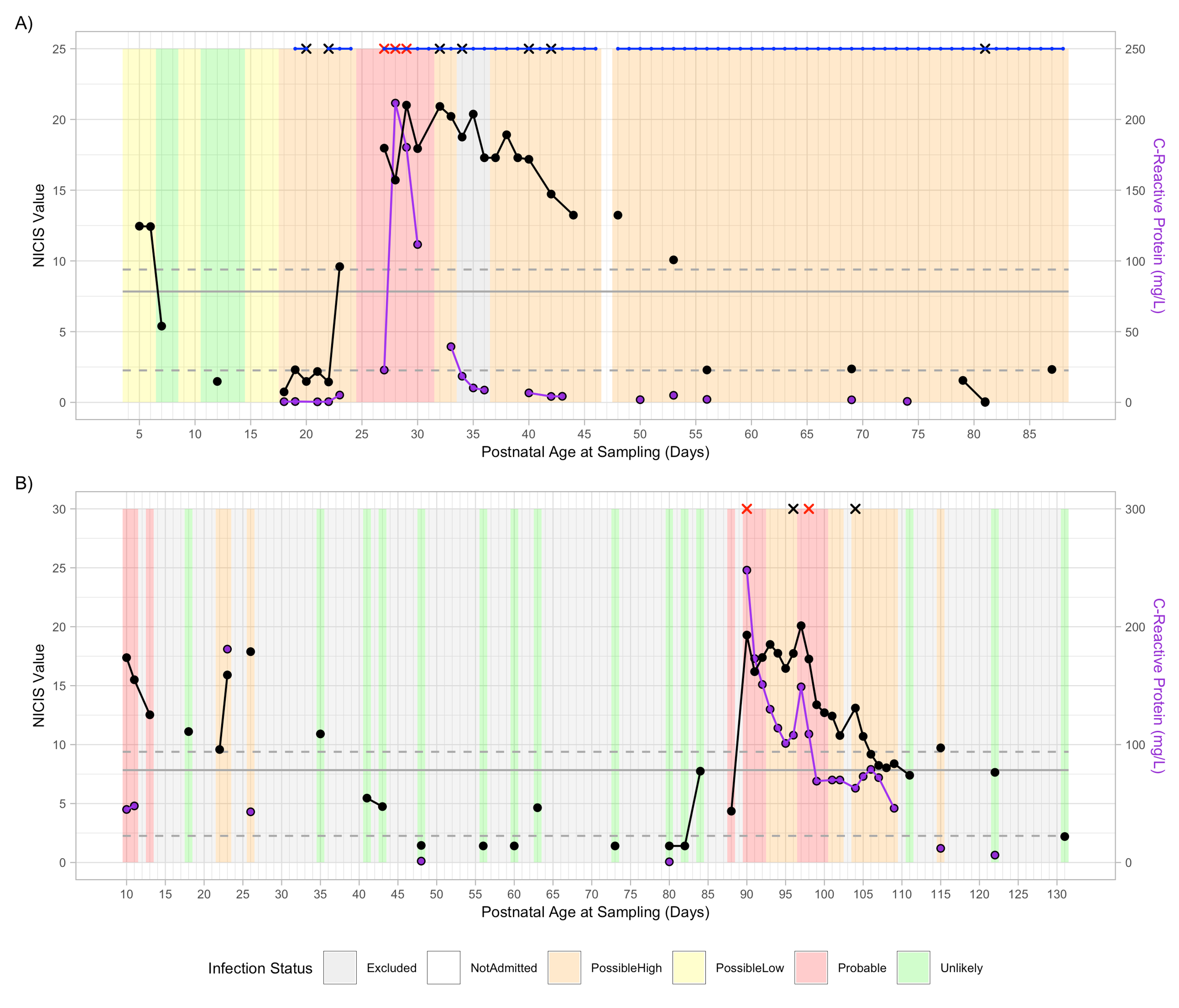

Supplement: Supplementary file 1 — Supplementary Fig. 1: Case studies illustrating the longitudinal utility of the Neonatal Intensive Care Infection Score (NICIS) compared to C-reactive protein (CRP). (A) Case study of a neonate born at 30 weeks’ gestation and admitted to the John Radcliffe Neonatal Intensive Care Unit. On days 27, 28, and 29, their blood cultures tested positive for Serratia marcescens. (B) Case study of a neonate born at 26 weeks’ gestation and admitted to the Erasmus University Medical Center Neonatal Intensive Care Unit. On days 90 and 98, their blood cultures tested positive for Klebsiella pneumoniae. NICIS values (black) and CRP measurements (purple) with antibiotic administration (blue lines and dots), positive/negative blood cultures (red/black crosses), NICIS thresholds for 95% sensitivity and 95% specificity (dashed grey line), and the optimal NICIS threshold (solid grey line) [file 44253_2026_121_MOESM1_ESM.png]
