# Supplementary material for: Development and validation of a diagnostic model for late-onset neonatal sepsis using the haematological profile: a retrospective cohort study
Source: Intensive Care Med Paediatr Neonatal. 2026 May 6;4(1):13. doi: 10.1007/s44253-026-00121-9 (PMC13314925; doi:10.1007/s44253-026-00121-9)
Supplement: Supplementary file 2 — Supplementary Material 2 [file 44253_2026_121_MOESM2_ESM.docx]

# Supplementary Tables

Supplementary Table 1: R packages used for data processing and analysis.

| Package | Version | Reference |
| --- | --- | --- |
| *tidyverse* | 2.0.0 | Wickham, H., Averick, M., Bryan, J., et al. (2019). tidyverse: Easily Install and Load the ‘Tidyverse’. https://CRAN.R-project.org/package=tidyverse |
| *rstatix* | 0.7.2 | Kassambara, A. (2023). rstatix: Pipe-Friendly Framework for Basic Statistical Tests. https://CRAN.R-project.org/package=rstatix |
| *bestNormalize* | 1.9.1 | Peterson, R. A. and Cavanaugh, J. E. (2020). bestNormalize: Normalizing Transformations. https://CRAN.R-project.org/package=bestNormalize |
| *pROC* | 1.18.4 | Xavier, R., Natacha, R., Alexandre, L., et al. (2011). pROC: Display and Analyze ROC Curves. https://CRAN.R-project.org/package=pROC |
| *epiR* | 2.0.84 | Stevenson, M. and Sergeant, E. (2025). epiR: Tools for the Analysis of Epidemiological Data. https://CRAN.R-project.org/package=epiR |
| *readxl* | 1.4.2 | Wickham, H., and Bryan, J. (2025). readxl: Read Excel Files. https://CRAN.R-project.org/package=readxl |
| *writexl* | 1.4.2 | Ooms, J. (2023). writexl: Export Data Frames to Excel. https://CRAN.R-project.org/package=writexl |
| *gridExtra* | 2.3 | Auguie, B. (2017). gridExtra: Miscellaneous Functions for ‘Grid’ Graphics. https://CRAN.R-project.org/package=gridExtra |

Supplementary Table 2: Diagnostic performance of individual complete blood count parameters for late-onset neonatal sepsis. Parameters highlighted in orange were included in the initial diagnostic model, and parameters highlighted in green were included in the final Neonatal Intensive Care Infection Score (NICIS). Performance metrics included area under the curve (AUC), true positives (TP), true negatives (TN), false positives (FP), false negatives (FN), positive predictive value (PPV), negative predictive value (NPV), positive likelihood ratio (LR+), negative likelihood ratio (LR-), and diagnostic odds ratio (DOR). Abbreviations: ch – channel units from the scattergram; FI – fluorescence intensity; SI – scatter intensity.

| Parameter | Units | P-Value | Effect Size | AUC | Threshold | Sens | Spec | TP | TN | FP | FN | PPV | NPV | LR+ | LR- | DOR | Youden |
| --- | --- | --- | --- | --- | --- | --- | --- | --- | --- | --- | --- | --- | --- | --- | --- | --- | --- |
| Red blood cell count | x10^12^/L | 0.02 | -0.25 | 0.588 | < 3.23 | 77.6% | 45.6% | 177 | 73 | 87 | 51 | 0.67 | 0.59 | 1.43 | 0.49 | 2.91 | 0.23 |
| Haemoglobin concentration | g/L | 0.02 | 0.12 | 0.569 | < 104 | 80.7% | 44.4% | 184 | 71 | 89 | 44 | 0.67 | 0.62 | 1.45 | 0.43 | 3.34 | 0.25 |
| Haematocrit | % | 0.02 | 0.12 | 0.572 | < 28.2 | 87.7% | 37.5% | 200 | 60 | 100 | 28 | 0.67 | 0.68 | 1.40 | 0.33 | 4.29 | 0.25 |
| Mean cell volume | fL | 0.07 | 0.19 | 0.552 | > 96.4 | 75.4% | 35.0% | 172 | 56 | 104 | 56 | 0.62 | 0.50 | 1.16 | 0.70 | 1.65 | 0.10 |
| Mean cell haemoglobin | pg | 0.09 | 0.18 | 0.545 | > 36.8 | 92.1% | 21.9% | 210 | 35 | 125 | 18 | 0.63 | 0.66 | 1.18 | 0.36 | 3.27 | 0.14 |
| Mean cell haemoglobin concentration | g/L | 0.34 | 0.10 | 0.530 | > 368 | 76.8% | 36.3% | 175 | 58 | 102 | 53 | 0.63 | 0.52 | 1.20 | 0.64 | 1.88 | 0.13 |
| Red blood cell distribution width | fL | < 0.001 | -0.46 | 0.624 | < 52.7 | 80.3% | 40.0% | 183 | 64 | 96 | 45 | 0.66 | 0.59 | 1.34 | 0.49 | 2.71 | 0.20 |
|  | % | < 0.001 | -0.74 | 0.710 | < 16.9 | 75.4% | 58.8% | 172 | 94 | 66 | 56 | 0.72 | 0.63 | 1.83 | 0.42 | 4.37 | 0.34 |
| Nucleated red blood cell count | x10^9^/L | < 0.001 | 0.33 | 0.694 | < 0.17 | 61.0% | 69.4% | 139 | 111 | 49 | 89 | 0.74 | 0.56 | 1.99 | 0.56 | 3.54 | 0.30 |
|  | % | < 0.001 | 0.26 | 0.650 | < 0.6 | 73.3% | 51.3% | 167 | 82 | 78 | 61 | 0.68 | 0.57 | 1.50 | 0.52 | 2.88 | 0.25 |
| Reticulocyte count | x10^12^/L | < 0.001 | 0.30 | 0.586 | > 0.130 | 80.2% | 39.6% | 182 | 63 | 96 | 45 | 0.65 | 0.58 | 1.33 | 0.50 | 2.65 | 0.20 |
|  | % | < 0.001 | 0.37 | 0.602 | > 4.3 | 87.7% | 34.0% | 199 | 54 | 105 | 28 | 0.65 | 0.66 | 1.33 | 0.36 | 3.66 | 0.22 |
| Immature reticulocyte fraction | % | 0.09 | 0.18 | 0.542 | > 16.7 | 20.7% | 91.2% | 47 | 145 | 14 | 180 | 0.77 | 0.45 | 2.35 | 0.87 | 2.70 | 0.12 |
| Red blood cell haemoglobin equivalent | pg | < 0.001 | 0.40 | 0.626 | > 31.0 | 74.9% | 50.9% | 170 | 81 | 78 | 57 | 0.69 | 0.59 | 1.53 | 0.49 | 3.10 | 0.26 |
| Reticulocyte haemoglobin equivalent | pg | < 0.001 | 0.72 | 0.703 | > 29.6 | 77.5% | 56.6% | 176 | 90 | 69 | 51 | 0.72 | 0.64 | 1.79 | 0.40 | 4.50 | 0.34 |
| Haemoglobin equivalent difference | pg | < 0.001 | 0.39 | 0.615 | > -2.4 | 56.4% | 64.8% | 128 | 103 | 56 | 99 | 0.70 | 0.51 | 1.60 | 0.67 | 2.38 | 0.21 |
| Hypochromic red blood cell count | % | < 0.001 | -0.42 | 0.619 | < 1.4 | 76.7% | 45.3% | 174 | 72 | 87 | 53 | 0.67 | 0.58 | 1.40 | 0.52 | 2.72 | 0.22 |
| Hyperchromic red blood cell count | % | 0.92 | -0.01 | 0.521 | < 0.8 | 64.3% | 46.5% | 146 | 74 | 85 | 81 | 0.63 | 0.48 | 1.20 | 0.77 | 1.57 | 0.11 |
| Microcytic red blood cell count | % | < 0.001 | -0.40 | 0.630 | < 4.3 | 62.3% | 66.9% | 142 | 107 | 53 | 86 | 0.73 | 0.55 | 1.88 | 0.56 | 3.33 | 0.29 |
| Macrocytic red blood cell count | % | < 0.001 | -0.28 | 0.582 | < 4.3 | 73.7% | 46.9% | 168 | 75 | 85 | 60 | 0.66 | 0.56 | 1.39 | 0.56 | 2.47 | 0.21 |
| White blood cell count | x10^9^/L | < 0.001 | -0.79 | 0.721 | < 15.26 | 50.4% | 88.8% | 115 | 142 | 18 | 113 | 0.86 | 0.56 | 4.48 | 0.56 | 8.03 | 0.39 |
| Neutrophil count | x10^9^/L | < 0.001 | 0.50 | 0.796 | < 5.46 | 70.6% | 78.8% | 161 | 126 | 34 | 67 | 0.83 | 0.65 | 3.32 | 0.37 | 8.90 | 0.49 |
|  | % | < 0.001 | -1.17 | 0.806 | < 45.5 | 72.4% | 77.5% | 165 | 124 | 36 | 63 | 0.82 | 0.66 | 3.22 | 0.36 | 9.02 | 0.50 |
| Lymphocyte count | x10^9^/L | < 0.001 | 0.59 | 0.659 | > 3.84 | 62.1% | 64.4% | 141 | 103 | 57 | 86 | 0.71 | 0.54 | 1.74 | 0.59 | 2.96 | 0.26 |
|  | % | < 0.001 | 1.34 | 0.836 | > 30.5 | 71.8% | 85.0% | 163 | 136 | 24 | 64 | 0.87 | 0.68 | 4.79 | 0.33 | 14.44 | 0.57 |
| Monocyte count | x10^9^/L | < 0.001 | -0.67 | 0.687 | < 2.70 | 43.9% | 90.0% | 100 | 144 | 16 | 128 | 0.86 | 0.53 | 4.39 | 0.62 | 7.03 | 0.34 |
|  | % | 0.40 | -0.09 | 0.523 | < 21.0 | 18.9% | 91.3% | 43 | 146 | 14 | 185 | 0.75 | 0.44 | 2.16 | 0.89 | 2.42 | 0.10 |
| Eosinophil count | x10^9^/L | 0.01 | 0.13 | 0.576 | > 0.20 | 42.1% | 85.0% | 96 | 136 | 24 | 132 | 0.80 | 0.51 | 2.81 | 0.68 | 4.12 | 0.27 |
|  | % | < 0.001 | 0.51 | 0.658 | > 2.1 | 50.0% | 84.4% | 114 | 135 | 25 | 114 | 0.82 | 0.54 | 3.20 | 0.59 | 5.40 | 0.34 |
| Basophil count | x10^9^/L | < 0.001 | 0.24 | 0.640 | < 0.06 | 85.5% | 40.0% | 195 | 64 | 96 | 33 | 0.67 | 0.66 | 1.43 | 0.36 | 3.94 | 0.26 |
|  | % | < 0.001 | -0.32 | 0.591 | < 0.7 | 34.2% | 80.6% | 77 | 129 | 31 | 151 | 0.71 | 0.46 | 1.77 | 0.82 | 2.16 | 0.15 |
| Immature granulocyte count | x10^9^/L | < 0.001 | -0.92 | 0.745 | < 0.19 | 64.0% | 76.3% | 146 | 122 | 38 | 82 | 0.79 | 0.60 | 2.70 | 0.47 | 5.72 | 0.40 |
|  | % | < 0.001 | -0.80 | 0.723 | < 1.4 | 69.3% | 68.8% | 158 | 110 | 50 | 70 | 0.76 | 0.61 | 2.22 | 0.45 | 4.97 | 0.38 |
| Neutrophil lymphocyte ratio | % | < 0.001 | -1.28 | 0.825 | < 1.46 | 76.7% | 76.3% | 174 | 122 | 38 | 53 | 0.82 | 0.70 | 3.23 | 0.31 | 10.54 | 0.53 |
| Immature to total neutrophil ratio | % | < 0.001 | -0.37 | 0.611 | < 0.06 | 36.4% | 83.8% | 83 | 134 | 26 | 145 | 0.76 | 0.48 | 2.24 | 0.76 | 2.95 | 0.20 |
| Neutrophil reactive intensity | FI | < 0.001 | -1.25 | 0.812 | < 46.1 | 75.0% | 74.4% | 171 | 119 | 41 | 57 | 0.81 | 0.68 | 2.93 | 0.34 | 8.71 | 0.49 |
| Neutrophil granularity intensity | SI | < 0.001 | 0.34 | 0.598 | > 144.2 | 40.4% | 76.9% | 92 | 123 | 37 | 136 | 0.71 | 0.47 | 1.75 | 0.78 | 2.25 | 0.17 |
| Neutrophil true width | ch | < 0.001 | -1.17 | 0.811 | < 37.3 | 68.4% | 81.9% | 156 | 131 | 29 | 72 | 0.84 | 0.65 | 3.78 | 0.39 | 9.79 | 0.50 |
| Reactive lymphocyte count | %L | < 0.001 | -0.61 | 0.675 | < 14.2 | 49.6% | 77.5% | 113 | 124 | 36 | 115 | 0.76 | 0.52 | 2.20 | 0.65 | 3.38 | 0.27 |
| Antibody-synthesising lymphocyte count | %L | < 0.001 | 0.26 | 0.649 | < 1.0 | 32.5% | 93.8% | 74 | 150 | 10 | 154 | 0.88 | 0.49 | 5.19 | 0.72 | 7.21 | 0.26 |
| High fluorescing lymphocyte count | %L | < 0.001 | 0.33 | 0.696 | < 0.0 | 41.0% | 91.9% | 95 | 144 | 16 | 132 | 0.86 | 0.52 | 5.05 | 0.64 | 7.85 | 0.33 |
| Reactive monocyte count | %M | < 0.001 | 0.28 | 0.667 | < 0.9 | 47.4% | 82.5% | 108 | 132 | 28 | 120 | 0.79 | 0.52 | 2.71 | 0.64 | 4.24 | 0.30 |
| Platelet count | x10^9^/L | < 0.001 | 0.87 | 0.731 | > 257 | 65.4% | 75.6% | 149 | 121 | 39 | 79 | 0.79 | 0.61 | 2.68 | 0.46 | 5.85 | 0.41 |
| Platelet distribution width | % | < 0.001 | -0.55 | 0.656 | < 13.7 | 71.0% | 57.0% | 98 | 81 | 61 | 40 | 0.62 | 0.67 | 1.65 | 0.51 | 3.25 | 0.28 |
| Mean platelet volume | fL | < 0.001 | -0.47 | 0.640 | < 11.5 | 69.6% | 54.9% | 96 | 78 | 64 | 42 | 0.60 | 0.65 | 1.54 | 0.55 | 2.79 | 0.25 |
| Platelet large cell ratio | % | < 0.001 | -0.51 | 0.646 | < 35.2 | 73.9% | 50.7% | 102 | 72 | 70 | 36 | 0.59 | 0.67 | 1.50 | 0.51 | 2.91 | 0.25 |
| Plateletcrit | % | < 0.001 | 0.52 | 0.633 | > 0.3 | 44.9% | 80.3% | 62 | 114 | 28 | 76 | 0.69 | 0.60 | 2.28 | 0.69 | 3.32 | 0.25 |
| Immature platelet fraction | % | < 0.001 | -0.91 | 0.740 | < 7.5 | 84.6% | 52.8% | 192 | 84 | 75 | 35 | 0.72 | 0.71 | 1.79 | 0.29 | 6.14 | 0.37 |
| Platelet to lymphocyte ratio | % | < 0.001 | 0.40 | 0.610 | > 0.059 | 52.0% | 70.0% | 118 | 112 | 48 | 109 | 0.71 | 0.51 | 1.73 | 0.69 | 2.53 | 0.22 |

Supplementary Table 3: Example calculation of NICIS: for each parameter, determine which threshold is crossed (optimal, 85% specificity, or 95% specificity), assign the corresponding AUC-weighted score, and sum the scores to calculate the total NICIS

| Parameter | Units | Result | Threshold Crossed | AUC | Score |
| --- | --- | --- | --- | --- | --- |
| Reticulocyte haemoglobin equivalent (RET-HE) | pg | 31.40 | None | 0.703 | 0.000 |
| Neutrophil count (NEUT#) | x10^9^/L | 7.52 | 85% Specificity | 0.796 | 1.592 (2 x AUC) |
| Immature granulocyte count (IG#) | x10^9^/L | 1.57 | 95% Specificity | 0.745 | 2.980 (4 x AUC) |
| Neutrophil lymphocyte ratio (NLR) | % | 2.38 | 85% Specificity | 0.825 | 1.650 (2 x AUC) |
| Neutrophil reactive intensity (NEUT-RI) | FI | 54.01 | 95% Specificity | 0.812 | 3.248 (4 x AUC) |
| Neutrophil true width (NEUT-TW) | ch | 72.79 | 95% Specificity | 0.811 | 3.244 (4 x AUC) |
| Platelet count (PLT#) | x10^9^/L | 198.65 | 85% Specificity | 0.731 | 1.462 (2 x AUC) |
| Immature platelet fraction (IPF) | % | 10.74 | Optimal | 0.740 | 1.480 (1 x AUC) |
|  |  |  | Total Score | | 15.656 |
|  |  |  | Likelihood of Late-Onset Sepsis | | *Probable*  (95% specificity) |

Supplementary Table 4: Paired NICIS and CRP measurements on probable sepsis days from the internal dataset, stratified by which test was measured first and whether each value exceeded its threshold. NICIS values were considered abnormal if above the optimal threshold (7.84), and CRP values were considered abnormal if above 10 mg/L.

| Pair Category | Number of Pairs | CRP Measured Before NICIS | NICIS Measured Before CRP | Median Time Between Results (mins; IQR) |
| --- | --- | --- | --- | --- |
| All pairs | 126 | 112 | 14 | - |
| NICIS abnormal/CRP abnormal | 59 | 5 | 4 | 35 (23 – 99) |
| NICIS abnormal/CRP normal | 46 | 37 | 9 | 36 (8 – 91) |
| NICIS normal/CRP abnormal | 8 | 7 | 1 | 32 (19 – 37) |
| NICIS normal/CRP normal | 13 | 13 | - | 60 (35 – 109) |
